# Prevalence and Determinants of *Schistosoma mansoni* Infection among Pre-School Age Children in Southern Ethiopia

**DOI:** 10.3390/pathogens12070858

**Published:** 2023-06-21

**Authors:** Tafese Tadele, Ayalew Astatkie, Solomon Mequanente Abay, Birkneh Tilahun Tadesse, Eyasu Makonnen, Eleni Aklillu

**Affiliations:** 1 School of Public Health, College of Medicine and Health Sciences, Hawassa University, Hawassa P.O. Box 1560, Ethiopia; 2 Department of Pharmacology and Clinical Pharmacy, College of Health Sciences, Addis Ababa University, Addis Ababa P.O. Box 9086, Ethiopia; 3 Department of Pediatrics, College of Medicine and Health Sciences, Hawassa University, Hawassa P.O. Box 1560, Ethiopia; 4 Center for Innovative Drug Development and Therapeutic Trials for Africa, College of Health Sciences, Addis Ababa University, Addis Ababa P.O. Box 9086, Ethiopia; 5 Department of Global Public Health, Karolinska Institutet, Karolinska University Hospital, 171 77 Stockholm, Sweden

**Keywords:** *Schistosoma mansoni*, neglected tropical diseases, public health problem, mass drug administration, pre-school age children, prevalence, Southern Ethiopia

## Abstract

School-based deworming program is implemented to control and eliminate *Schistosoma mansoni* infection in many endemic countries, including Ethiopia. However, pre-school-age children (pre-SAC) are not targeted to receive preventive chemotherapy against *S. mansoni* infection, partly due to a lack of information on the disease burden. We assessed the prevalence and correlates of *S. mansoni* infection among pre-SAC in Southern Ethiopia. A total of 1683 pre-SAC aged 4 to 7 years were screened for *S. mansoni* infection. A multilevel binary logistic regression was fitted to detect the significant determinants of *S. mansoni* infection. Adjusted odds ratios (AORs) with a 95% confidence interval (CI) were used to identify determinants of *S. mansoni* infection. The overall prevalence of *S. mansoni* infection was 14.3% (95% CI: 12.6, 16.0%). *S. mansoni* infection was significantly higher among 6-year-old (AOR = 2.58, 95% CI: 1.55, 4.27) and 7-year-old children (AOR = 4.63, 95% CI: 2.82, 7.62). Accompanying others to water sources sometimes (AOR = 2.60, 95% CI: 1.12, 6.01) and all the time (AOR = 5.91, 95% CI: 2.51, 13.90), and residing in less than one kilometer from the infested water source (AOR = 3.17, 95% CI: 1.47, 6.83) increased the odds of *S. mansoni* infection. In conclusion, the prevalence of *S. mansoni* infection among pre-SAC in the study area was moderate. The study highlights the urgent need to include pre-SAC aged 4 to 7 years in annual preventive chemotherapy campaigns to reduce the risk of possible sources of infection and enhance the achievement of the elimination target.

## 1. Introduction

Schistosomiasis is one of the prominent neglected tropical diseases causing major public health threats in different parts of the world [1]. It affects mostly people living in the tropics and subtropics, predominantly the deprived and utmost poor societies [2,3]. In schistosomiasis endemic areas, the most dominant form is chronic schistosomiasis, resulting from repeated exposure to the infectious larval stage [4,5]. 

Children are often infected at the age of 2 years, and many of them remain chronically infected throughout their school-age years [6]. During pre-school age (pre-SAC), the exposure to infection is progressive, and practically all children in hyper-endemic areas can be exposed to infective cercariae at one year of age [7,8]. Once individuals are infected with *S. mansoni* parasites, they are at risk of acquiring related morbidity [9,10,11,12,13]. *S. mansoni* morbidity is initiated principally by injurious immunologic responses to *S. mansoni* eggs deposited in the liver and intestine by adult female worms living in the vasculature surrounding these organs [14,15]

The burden of *S. mansoni* infection shows wide geographical variations across endemic countries in Sub-Saharan Africa (SSA). Some studies reported a higher prevalence of *S. mansoni* infection in pre-SAC ranging from 11.8% to 70.5% [16,17,18,19,20,21], while some reported a lower rate ranging from 0.9% to 5.9% [15,22,23]. Small-scale studies reported that daily exposure to infested water bodies and high levels of cumulative water contact in habitats where snail hosts are found is linked with the risk of transmission among pre-SAC [8,24,25,26]. Moreover, children’s habit of swimming as well as the frequency of swimming in rivers and children’s habit of crossing rivers barefoot are associated with intestinal schistosomiasis in Ethiopia [27,28]. 

The revised World Health Organization (WHO) roadmap targets the elimination of schistosomiasis as a public health problem by 2030 and the interruption of schistosome transmission in selected countries by 2030 [29]. One of the WHO-recommended public health interventions to interrupt transmission is the large-scale periodic administration of praziquantel to all at-risk populations as preventive chemotherapy (PC) [30]. Ethiopia is among the high schistosomiasis burden countries in SSA with recent surveys reporting prevalence levels between 24 to 76.3% among school-age children (SAC) [31]. The disease prevalence varies across the country, the southern region being the most affected (40%) [32]. The Ethiopian neglected tropical diseases (NTDs) program initiated school-based PC in 2014 primarily targeting SAC to control schistosomiasis [33]. Despite substantial progress in reducing the overall burden, a recent study conducted after several rounds of PC implementation among SAC reported an overall *S. mansoni* infection prevalence of 25.8% (range between schools 11.6% to 54.1%) in Southern Ethiopia [3]. 

Expansion of preventive chemotherapy to all in need, including pre-SAC, and a single prevalence threshold to conduct preventive chemotherapy and its frequency is among the recent six evidence-based recommendations by WHO for the elimination of morbidity and interruption of disease transmission [34]. The Ethiopian National NTD control program aims to eliminate schistosomiasis from being public health problem by 2025 defined as a prevalence of schistosomiasis of <2%, or maintain <1% prevalence of heavy infection in at-risk populations [35]. Currently, the national NTD program implements school-based targeted PC with praziquantel, and the frequency of PC is based on prevalence data. Pre-SAC is not yet included in the PC program. Epidemiological studies are imperative to provide evidence-based recommendations for policymakers on whether to include pre-SAC in PC campaigns and determine its frequency. Therefore, we conducted this study to generate evidence on the magnitude and potential determinants of *S. mansoni* infection to guide the inclusion of pre-SAC in the mass drug administration (MDA) program in Southern Ethiopia. 

## 2. Materials and Methods

### 2.1. Study Area, Population, and Design

**Figure 1:**
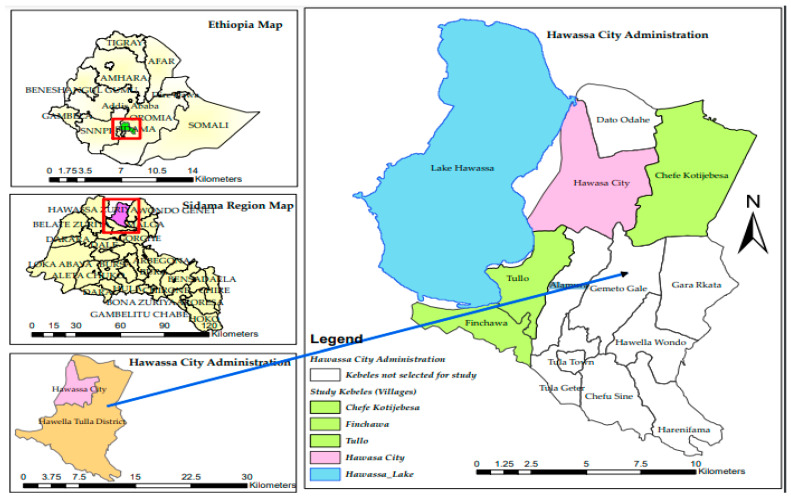
Map of the study site. The (**top** left) is the map of Ethiopia. The (**middle** left) figure shows the map of the Sidama region, and the (**bottom** left) figure indicates the map of Hawassa City administration where the study districts are located. The figure on the right shows the map of Lake Hawassa and the villages where the study was conducted.

A cross-sectional study was conducted from August to December 2021 among pre-SAC (children aged 4 to 7 years) in the Hawella Tulla district of Sidama region, Southern Ethiopia. The study district was found to have a higher prevalence of schistosomiasis as per the general mapping exercise of NTDs conducted in Ethiopia between 2013 and 2014 [36]. Hawella Tulla district is located along the shore of Lake Hawassa and Tikur Wuha River at 289 km south of Addis Ababa, the capital city of Ethiopia. It is situated at an altitude of 1800 m above sea level and has an annual rainfall of 1123 mm and a temperature of 13–27 °C. Lake Hawassa is the water source for the residents of Hawella Tulla for their domestic, agricultural, fishing, and other uses. A map of the study district is presented in Figure 1.

### 2.2. Eligibility Criteria

Children aged 4 to 7 years who resided at least six months in the selected villages of Hawella Tulla district before the study were included. Pre-SAC who were treated with praziquantel within a month of the study recruitment period based on the verbal information obtained from the mothers/primary caregivers were excluded for the parasites could be cleared because of prior treatment. Children who were sick or with severe medical conditions and unable to produce stool samples were also excluded. 

### 2.3. Sample Size Determination

OpenEpi (a free web-based open-source epidemiological statistics) was used to estimate the sample size for the assessment of the outcome and infection rate of *S. mansoni* in pre-SAC. The sample size was computed using the prevalence and risk factors to address its representativeness to the large population. The assumptions used were a prevalence (p) of 25% from a previous study in the same age group in Ethiopia [19], 3% absolute precision, and a design effect of 2. Accordingly, the minimum sample size (n) was found to be 1600, and the total sample size was 1778 after adjustment for a 10% nonresponse rate. The sample size needed to identify the risk factors associated with *S. mansoni* infection was calculated considering variables significantly associated with *S. mansoni* infection in previous studies and fixing the level of confidence at 95%, power at 90%, the ratio of unexposed-to-exposed at 1% and anticipated nonresponse rate at 10%. The sample sizes calculated for risk factors using the findings of the previous report were then 138, 134, and 214. Hence, the sample size calculated from the prevalence (n = 1778) was used as the final sample size for the present study as it would meet all objectives of the study.

### 2.4. Sampling Procedure

A multistage sampling technique was used to recruit the study participants. Hawella Tulla district has twelve kebeles (lowest administrative units in Ethiopia, hereafter referred to as village(s)), of which, six were selected for this study based on the *S. mansoni* infection rate in school-age children as per the mapping study of the Ethiopian Public Health Institute in collaboration with the Ministry of Health of Ethiopia [36]. In the first stage, three villages (Tullo, Finchawa, and Chefe Kotijebesa) were selected by simple random sampling technique from six villages. In the selected villages, the sampling frame of the eligible study participants was prepared through house-to-house enumeration by health extension workers before the commencement of actual data collection. Whenever more than one eligible child was found in the same selected household, only one was included in the sampling frame using the lottery method. In the second sampling stage, the number of study participants included in each village was determined by proportional allocation based on the total number of participants found in each village. Then, the study participants were selected by a simple random sampling method. At times when the sampled child was absent from home, two repeated visits (one out of marketing days and one during the weekend) were made. Those pre-SAC who were not traced after three visits were considered non-respondents. 

### 2.5. Study Variables

The outcome variable for this study was *S. mansoni* infection which was detected using Kato–Katz (a gold standard method) for diagnosing *S. mansoni* by microscopic examination [30,37]. This takes a binary outcome, such that *S. mansoni* infection was regarded as infected (1 = if *S. mansoni* infection detected) or uninfected (0 = if no *S. mansoni* infection detected). Egg counts were quantified and classified for *S. mansoni* infection according to the WHO guideline as light (1–99 EPG), moderate (100–399 EPG), or heavy (>400 EPG) [30,37].

The exposure variables included age, sex, educational level, and occupation of the mothers/primary caregivers of pre-SAC, sanitary practices, water contact behavior, distance from the infested water body, latrine use, and the wealth index of the household. The age of the study participant was determined by asking about their last birthday. The wealth index of the household was constructed from the household asset and its interquartile range was fixed. The distance from the infested water body was categorized according to the predetermined classification of the distance (less than 1 km, 1 to 2 km, and greater than 2 km) using the GPS before the actual data collection period.

### 2.6. Data Collection Tools and Procedures

Data were collected using a pre-tested structured Amharic or *Sidaamu Afoo* (the local language) version questionnaire using an interview technique. Eight research assistants with public health expertise who were trained on how to apply the questionnaire interviewed the mothers/primary caregivers of the participants. The interview was conducted at the respondent’s home. The questionnaire was prepared in English, translated into Amharic and *Sidamu Afoo* languages, and translated back to English to check the consistency. The Amharic or *Sidamu Afoo* questionnaire was used for interviews based on the language proficiency of the respondents and their preferences. Two senior experts and the principal investigator supervised the data collection process. 

A stool specimen was collected from each study participant using a wide-mouth 100 mL screw-capped dry and clean container pre-labeled with the participant’s unique identification number. The collected stool samples were transported within an hour of collection in suitable cool boxes at temperatures between 4 and 60c for subsequent examination at Bushulo health center. Samples were examined using the Kato–Katz technique for the detection of parasite eggs, with two slides per stool. A portion of the sample was processed by the Kato–Katz method using a template holding 41.7 mg of stool [32]. Two experienced laboratory technologists examined the slides, and the difference in the test results was managed using the third experienced laboratory technologist. The number of eggs was counted and multiplied by 24 to obtain the number of EPG of stool.

The presence of *S. mansoni*, *Ascaris lumbricoides, Trichuris trichuria*, and hookworm eggs was recorded. Examination for hookworm infection was performed within an hour of smear preparation, while examination for *S. mansoni, A. lumbricoides, and T. trichuria* was performed within 24 h after smear preparation. 

### 2.7. Data Analysis

Data were recorded on standard record forms, entered into the RedCap database, and exported to an Excel file for cleaning. The data analysis was conducted using Stata software version 14 [StataCorp LLC, College Station, TX, USA]. Descriptive analyses were conducted by calculating frequencies and percentages for categorical variables and mean with standard deviations and medians with interquartile ranges. The determinants of *S. mansoni* infection were investigated using multilevel logistic regression.

The suitability of the data for standard logistic regression versus multilevel logistic regression was checked by fitting an intercept-only model and comparing it with the null model from a standard logistic regression. An intra-class correlation coefficient (ICC) of 0.084 from the intercept-only model and a significant likelihood ratio test (LR) with a corresponding *p*-value less than 0.005, and lower Akaike’s information criterion (AIC) and Bayesian information criterion (BIC) for the intercept-only model vis-à-vis the null model from standard logistic regression showed the need for multilevel analysis. Hence, multilevel logistic regression was applied to identify the determinants of *S. mansoni* infection as it provides a more accurate description of relationships in clustered data by correcting underestimated standard errors, estimating components of variance at several levels, and estimating cluster-specific intercepts and slopes than standard logistic regression. 

To conduct a multilevel binary logistic regression analysis, three models were fitted. The first model was the null model (a model without the explanatory variables), which shows the extent of variability in *S. mansoni* infection due to cluster- (village-) level effect without accounting for covariate effects. The second model, model I, comprised individual-level determinants with random intercept. The third model, model II, which included individual-level predictors with random intercept and random-coefficient for the distance of the household from an infested water source, was the final model. An interaction effect was checked between the age and sex of the participants. However, no significant interaction effect was detected. To identify the best-fitting model, comparison was conducted between model I and model II. The result revealed that there was no significant improvement in the fit of model II relative to model I based on the likelihood ratio test. The changes in AIC and BIC values were also minimal. However, the ICC increased by 177% (from 11.5% to 31.8%) after accounting for the random coefficient for the distance of the households from an infested water source in model II. In addition, the odds ratio for distance less than 1 km changed by about 26% from model I to model II. Furthermore, the variance of the coefficient of the distance of households from infested water sources in model II across village was significant. It is also logically plausible to assume that the effect of distance from infested water sources on the risk of *S. mansoni* infection varies across villages. Therefore, we took the third model, model II, as our final model. See supplementary file S1 (Table S1) for the details of the model comparison. The selection of variables for inclusion into the multivariable multilevel analysis was conducted based on a *p*-value < 0.25 from the crude analysis.

Based on the final model, adjusted odds ratios (AORs) with a 95% confidence interval (CIs) were reported based on the fixed-effects component of the model. See supplementary file S2 (Table S2) for results from all models. Random effects for the intercept and coefficients were reported based on variances across clusters with 95% CIs. Ninety-five percent of CIs of AORs that do not embrace 1 indicated statistically significant fixed effects, whereas 95% of CIs of variances that do not embrace 0 implied statistically significant village-level random effects.

## 3. Results

### 3.1. Study Participants’ Characteristics 

A total of 1683 (95%) pre-SAC aged 4 to 7 years participated in the study. Out of the total participants, 52.3% were males (*n* = 880). The mean (±standard deviation (SD)) age of the children was 5.49 (±1.09) years (Table 1). 

### 3.2. Prevalence of S. mansoni Infection 

A total of 241/1683 pre-SAC, 14.3%, (95% CI: 12.6%, 16.0%) were found to be infected with *S. mansoni*, with a prevalence of 16.4% (144/880) in males and 12.1% (97/803) in females. The prevalence of infection increased with increasing age; the youngest age group (4 years) exhibited a moderate prevalence of 6.9% and the much older age group (7 years) had a high prevalence of 24.7% (Table 2). The prevalence of infection varied by study villages. Among the three villages that participated in this study, Tullo village had the highest prevalence of *S. mansoni* infection (21.4%), followed by Finchawa village (10.5%), and Chefe Kotijebesa village (6.6%). Microscopic examinations of stool samples showed that about 73 (30.3%) *S. mansoni*-infected pre-SAC had multiple parasite infections.

### 3.3. Intensity of S. mansoni Infection in Pre-SAC

*S. mansoni* egg count in test stool of the 241 pre-SAC who were microscopy positive ranged from 12 to 2560 EPG. Most of the infected pre-SAC had a low egg count of 12 to 96 EPG (n = 137; 8.1% light infection). The remaining (n = 69; 4.1% moderate infection) pre-SAC had 108 to 396 EPG, while (n = 35) (2.1%; heavy infection) had 400 to 2560 EPG (Table 2). The heaviest infection intensity was detected from Tullo *kebele* (1.7%) followed by Chefe Kotijebesa *kebele* (0.3%), and Finchawa *kebele* (0.2%). 

### 3.4. Determinants of S. mansoni Infection in Pre-SAC

#### 3.4.1. Effect of Determinants on *S. mansoni* Infection 

In the multivariable multilevel logistic regression analysis, *S. mansoni* infection was significantly associated with higher age, i.e., a child aged 6 years (AOR = 2.58, 95% CI: 1.55, 4.27) and a child aged 7 years (AOR = 4.63, 95% CI: 2.82, 7.62). Accompanying others to water sources sometimes (AOR = 2.60, 95% CI: 1.12, 6.01) and all the time (AOR = 5.91, 95% CI: 2.51, 13.90), and living in less than 1 km radius from the nearby water body, Lake Hawassa, (AOR = 3.17, 95% CI: 1.47, 6.83) were also found to be the variables significantly associated with *S. mansoni* infection. The occupation and wealth index of the mothers/primary caregivers of pre-SAC had no association with *S. mansoni* infection (Table 3). 

#### 3.4.2. Between-Village Variability on the Risk of *S. mansoni* Infection 

There was significant village-level variability in the intercept of the model (variance = 0.30; 95% CI: 0.05, 1.65). Similarly, the effect of the distance of households from infested water sources on the risk of *S. mansoni* infection was variable across villages (variance = 0.077; 95% CI: 0.07653, 0.07654). Controlling for other covariates, 31.8% of the variability on the risk of *S. mansoni* infection was explained by village membership. Supplementary file S1 shows the details of the models.

## 4. Discussion

This study aimed to explore the burden of *S. mansoni* infection among pre-SAC in Southern Ethiopia and determine the factors associated with the infection. The present study found that the prevalence of *S. mansoni* infection among pre-SAC was 14.3%, with significant variation across villages. The prevalence was higher among children aged 6 and 7 years, those residing within one-kilometer radius of the nearby water body, and those who accompanied others to water sources all the time or sometimes. The effect of distance of households from infested water sources on the risk of infection varied across villages. Furthermore, village membership accounted for 31.8% of the variability in the risk of infection.

The burden of *S. mansoni* infection among pre-SAC significantly varies across geographical localities in endemic settings. Thus, the present study compared the results with a previous study among SAC in the same area [3], which reported an overall *S. mansoni* infection prevalence of 24.6%, as there was no previous study among pre-SAC in the study area. The overall *S. mansoni* infection prevalence of 14.3% reported in the present study was lower than the prevalence previously reported among SAC in the same area [3]. The difference in prevalence between the current study and the previous study among SAC may be attributed to the reduction of infection in the study area which resulted from the initiation of praziquantel MDA to SAC in 2014. There is evidence that treating SAC infected with schistosomiasis tends to lower the prevalence among the entire population by reducing the ongoing transmission of the infection [38]. However, the MDA among SAC was interrupted in 2015 and low treatment coverage was reported in 2016 which may have contributed to the spread of infection among pre-SAC in the study area. 

The prevalence and heavy-intensity infection of *S. mansoni* detected in the present study is above the national NTD program target to eliminate schistosomiasis [35]. Concerning the national NTD target, the current study highlights the urgent need to include pre-SAC in control and elimination interventions against schistosomiasis, as WHO recommends starting from the age of 2 years [38]. Moreover, the observed heavy-intensity infection rate in the most affected village among pre-SAC indicates that *S. mansoni* infection may continue to be a major public health threat in the study area. Hence, the WHO target to reach <1% prevalence of heavy infection intensity in children aged 5–14 years by 2030 [39], could be difficult to achieve. The authors suggest that the burden of this disease found in the present study and in the previous report of the SAC study indicate the need for the inclusion of pre-SAC in MDA, rather than only relying on and sustaining the involvement of SAC in schistosomiasis control and elimination programs in Ethiopia. Thus, the recently initiated Ethiopian Ministry of Health community-based deworming strategy that takes place integrated with a regular health extension program [40], would be the best way to reach pre-SAC once the praziquantel pediatric formulation becomes available.

The current study found that the odds of *S. mansoni* infection were higher among children aged 6 and 7 years compared to younger age groups. Older pre-SAC were observed to have more opportunities to go with their elder siblings and parents to the infested water source compared to younger pre-SAC. During the interview, the reported increased contact of contaminated stagnant water for playing purposes among older pre-SAC may contribute to the higher risk of infection. Additionally, the hot weather condition of the study area and the increased density of infected intermediate snail hosts in the water bodies, especially during the hot hours of the day, may favor the spread of *S. mansoni* infection among pre-SAC in the study area. 

The present study revealed accompanying others to water sources sometimes and all the time was a significant determinant of *S. mansoni* infection among pre-SAC. The reason for this association is that in rural parts of Ethiopia, it is common for pre-SAC to accompany their mothers/elder siblings to infested water bodies, which could expose them to acquiring the infection at a young age. Another explanation could be the fact that mothers or primary caregivers of the rural community may not give due attention to actively and closely monitoring pre-SAC compared to their urban counterparts, thus pre-SAC living around lake shore villages are potentially exposed to high risk of *S. mansoni* infection.

In the present study, children who are living within less than 1 km radius distance of an infested water body had three times more odds to be infected compared to those who are residing in greater than 2 km distance. A similar finding was previously reported among SAC in the same area [3], in schools found in villages located less than 1 km from infested water bodies. This might be associated with the frequency of infested water contact, low knowledge in prevention and control of *S. mansoni* infection among mothers/primary caregivers, and more engagement in predisposing practices among pre-SAC in the study area. 

The current study findings indicate that the effect of the distance of households from infested water sources on the risk of *S. mansoni* infection varied significantly across villages. Additionally, from our village-specific analysis of the prevalence of *S. mansoni* infection, children from villages closest to Lake Hawassa were at a higher risk of infection than children away from the lake. Moreover, the intensity of other risk factors such as frequency of contact with infested water may vary based on distance from the infested water source, which may in turn lead to differences in the effect of distance across villages. 

In the present study, 31.8% of the variability in *S. mansoni* infection was attributed to the village membership whereas between-individual variation explained the remaining 68.2% of the total variability. This indicates the existence of significant heterogeneity in the risk of *S. mansoni* infection across different villages due to differences in the chance of acquiring the infection among pre-SAC. The difference could be attributed to the differential proximity of the infested water source, increased water contact, socioeconomic status, and variation in the magnitude of snail hosts among the study villages. 

The study has some limitations. First, the Kato–Katz technique that we employed may underestimate the egg counts in an area with low prevalence. Second, the diarrheic consistency of the stool samples from study participants rendered the preparation of Kato–Katz slides difficult, which may result in an underestimation of the prevalence of *S. mansoni* infection. Third, the study did not assess the community-level determinants to investigate village-level predictors, which account for the village-level variability of *S. mansoni* infection among pre-SAC.

## 5. Conclusions

The prevalence of *S. mansoni* infection among pre-SAC in the schistosomiasis endemic Hawella Tulla district of Southern Ethiopia was moderate. The prevalence varies widely by age of the children, with those at the age of 6 and 7 years having the highest risk. The risk of infection also varies across villages. Further, the frequency of accompanying others to infested water sources and living at less than 1 km from infested water sources increased the risk of *S. mansoni* infection. The effect of distance on the risk of *S. mansoni* infection varies significantly across villages. Therefore, we recommend the inclusion of pre-SAC in annual PC integrated with potable water supplies, sanitation, and hygiene (WASH) interventions, and vector control to reduce the morbidity of chronic schistosomiasis and reduce transmission, which helps achieve the elimination target of schistosomiasis. Moreover, the provision of health education for behavior change to avoid contaminated water contact is recommended for all village communities. 

## Figures and Tables

**Table 1:** The individual characteristics of pre-school age children and their mothers/primary caregivers in Southern Ethiopia, August to December 2021.

Variable	Category	Frequency N (%)
Age (in years)	Four	393 (23.4)
	Five	465 (27.6)
	Six	424 (25.2)
	Seven	401 (23.8)
Sex	Female	803 (47.7)
	Male	880 (52.3)
Educational level of mothers/primary caregivers of pre-SAC	No formal education	701 (41.6)
	Primary education	486 (28.9)
	Secondary education	232 (13.8)
	College and above	264 (15.7)
Current major occupation of mothers/primary caregivers of pre-SAC	Farmer	397 (23.6)
	Full-time housewife	1139 (67.7)
	Merchant/employed	147 (8.7)
Wealth Index	Poorest	413 (24.5)
	Poorer	357 (21.2)
	Middle	271 (16.1)
	Richer	313 (18.6)
	Richest	329 (19.6)
District	Tullo	763 (45.3)
	Finchawa	447 (26.6)
	Chefe Kotijebesa	473 (28.1)
Distance from the water sources	Less than 1 km	304 (18.1)
	1 km to 2 km	977 (58.0)
	More than 2 km	402 (23.9)

N = total number of participants; Pre-SAC: pre-school age children; km: kilometer.

**Table 2:** Prevalence and intensity of *S. mansoni* infection by age groups among pre-school age children in Southern Ethiopia, August to December 2021.

Age	*S. mansoni* Infection Prevalence	*S. mansoni* Infection Intensity in Pre-SAC		
		Light	Moderate	Heavy
Overall	14.3%	8.1%	4.1%	2.1%
4 years	6.9%	4.1%	2.3%	0.5%
5 years	9.9%	5.5%	3.0%	1.3%
6 years	16.3%	9.9%	3.6%	2.8%
7 years	24.7%	13.2%	7.7%	3.8%

*S. mansoni*; *Schistosoma mansoni*; Pre-SAC: pre-school age children.

**Table 3:** Rates and determinants of *S. mansoni* infection among pre-school age children in Southern Ethiopia using multilevel logistic regression.

Variable	Category	*S. mansoni* Infection		Univariable Multilevel LR		Multivariable Multilevel LR	
		Non-Infected	Infected	COR (95%Cl)	*p* Value	AOR (95%Cl)	*p* Value
Age (in years)	Four	366 (93.1%)	27 (6.9%)	1 ^a^		1 ^a^	
	Five	419 (90.1%)	46 (9.9%)	1.62 (0.98, 2.68)	0.06	1.57 (0.93, 2.66)	0.09
	Six	355 (83.7%	69 (16.3%)	3.00 (1.86, 4.83)	<0.001	2.58 (1.55, 4.27)	<0.001
	Seven	302 (75.3%)	99 (24.7%)	5.72 (3.59, 9.12)	<0.001	4.63 (2.82, 7.62)	<0.001
Marital status of parents	Married	1412 (86.1)	228 (13.9)	1 ^a^		1 ^a^	
	Not married	30 (69.8)	13 (30.2)	2.28 (1.16,4.51)	0.02	1.40 (0.65, 3.01)	0.39
Sex	Female	706 (87.9)	97 (12.1))	1 ^a^		1 ^a^	
	Male	736 (83.6)	144 (16.4)	1.49 (1.12, 1.97)	0.006	1.25 (0.92, 1.70)	0.16
The educational level of mothers/primary caregivers of pre-SAC	College and above	235 (89.1)	29 (10.9)	1 ^a^		1 ^a^	
	No formal education	594 (84.7)	107 (15.3)	1.68 (1.08, 2.62)	0.02	1.18 (0.68, 2.02)	0.56
	Primary education	410 (84.4)	76 (15.6)	1.81 (1.13, 2.88)	0.01	1.46 (0.86, 2.49)	0.17
	Secondary education	203 (87.5)	29 (12.5)	1.24 (0.71, 2.17)	0.44	1.03 (0.56, 1.89)	0.92
Contact to infested water	No	676 (90.5)	71 (9.5)	1 ^a^		1 ^a^	
	Yes	766 (81.8)	170 (18.2)	2.16 (1.60, 2.92)	<0.001	0.71 (0.31, 1.60)	0.40
Frequency of accompany to the water source	Never	675 (91.5)	63 (8.5)	1 ^a^		1 ^a^	
	Sometime	631 (84.5)	116 (15.5)	1.96 (1.41, 2.72)	<0.001	2.60 (1.12, 6.01)	0.03
	All the time	136 (68.7)	62 (31.3)	5.05 (3.35, 7.63)	<0.001	5.91 (2.51, 13.90)	<0.001
Wealth index	Richest	283 (86.0)	46 (14.0)	1.0		1 ^a^	
	Poorest	361 (87.4)	52 (12.6)	1.08 (0.70, 1.68)	0.72	1.06 (0.62, 1.80)	0.84
	Poorer	304 (85.2)	53 (14.9)	1.35 (0.87,2.09)	0.18	1.10 (0.65, 1.85)	0.72
	Middle	227 (83.8)	44 (16.2)	1.64 (1.03, 2.60)	0.04	1.54 (0.90, 2.63)	0.11
	Richer	267 (85.3)	46 (14.7)	1.23 (0.78,1.93)	0.37	1.05 (0.63, 1.76)	0.85
Distance to the water sources	More than 2 km	356 (88.6)	46 (11.4)	1 ^a^		1 ^a^	
	Less than 1 km	215 (70.7)	89 (29.3)	3.50 (2.34, 5.24)	<0.001	3.17 (1.47, 6.83)	0.003
	1 km to 2 km	871 (89.2)	106 (10.9)	1.04 (0.72, 1.52)	0.81	1.04 (0.63, 1.72)	0.89

^a^: Reference category; LR: logistic regression; COR: crude odds ratio; AOR: adjusted odds ratio; CI: 95% confidence interval.

## Data Availability

All data presented in this study are contained within the manuscript.

## Supplementary Materials

The following supporting information can be downloaded at: https://www.mdpi.com/article/10.3390/pathogens12070858/s1, Table S1: Model comparison using multivariable multilevel logistic regression analysis among pre-school age children (Pre-SAC) infected with *S. mansoni* in Southern Ethiopia, August to December 2021; Table S2: Multilevel logistic regression analysis of determinants of *S. mansoni* infection among pre-school age children (Pre-SAC) in Southern Ethiopia, August to December 2021.
