# Supplementary material for: Prevalence and Determinants of Schistosoma mansoni Infection among Pre-School Age Children in Southern Ethiopia
Source: Pathogens. 2023 Jun 21;12(7):858. doi: 10.3390/pathogens12070858 (PMC10385345; doi:10.3390/pathogens12070858)
Supplement: Supplementary file 1 [file pathogens-12-00858-s001.zip › pathogens-2420343-supplementary.pdf]

Table S1: Multivariable multilevel logistic regression analysis of *S. mansoni* infection among pre-school age children (pre-SAC) in southern Ethiopia, August to December 2021

**Parameters**	**Null model with random intercept**	**Model I (Individual-level predictors with random intercept)**	**Model II (Individual-level predictors with random intercept and random-coefficient for distance of the households from infested water source)**
Model comparison			
Multilevel multivariable logistic regression analysis model			
Kebele level variance (95% CI)of the intercept	0.30 (0.05, 1.65)	0.43 (0.08, 2.30)	1.5358 (1.5357, 1.5359)
Kebele level variance (95% CI) of the coefficient of distance from infested water source	N/A	N/A	0.077 (0.07653, 0.07654)
Log-likelihood	-666.29	-573.73	-573.96
ICC %	8.4%	11.5%	31.8%
AIC	1336.59	1185.46	1189.92
BIC	1347.44	1288.60	1303.92

Null model: intercept only model (a model without the explanatory variables)

Model I: Model with individual-level predictors with random intercept

Model II: Model with individual-level predictors with random intercept and random-coefficient for distance of the households from water source

N/A: Not applicable; [a]ICC: Intra-class Correlation Coefficient; [a]AICAkaike's information criterion; [a]BIC Bayesian information criterion

Table S2: Multilevel logistic regression analysis of predictors associated with *S. mansoni* infection among pre-school age children (pre-SAC) in southern Ethiopia, August to December 2021

Individual-level determinants	Models		
	Null modelAOR (95%Cl)	Model I AOR (95%Cl)	Model II AOR (95%Cl)
Child age (years)			
4 years		1[a]	1[a]
5 years		1.54 (0.91, 2.60)	1.57 (0.93, 2.66)
6 years		2.59 (1.57, 4.28)***	2.58 (1.55, 4.27)***
7 years		4.69(2.86, 7.69)***	4.63 (2.82, 7.62)***
Marital status of mothers/primary caregivers			
Married		1[a]	1[a]
Not married		1.43 (0.67, 3.06)	1.40 (0.65, 3.01)
Sex			
Female		1[a]	1[a]
Male		1.24(0.91, 1.68)	1.25 (0 .92, 1.70)
Educational level of mothers/primary caregivers			
College and above		1[a]	1[a]
No formal education		1.16 (0.68, 1.98)	1.18(0.68, 2.02)
Primary education		1.46 (0.86, 2.49)	1.46 (0.86, 2.49)
Secondary education		1.01 (0 .55, 1.85)	1.03 (0.56, 1.89)
History of water contact			
No		1[a]	1[a]
Yes		0.71 (0.32, 1.59)	0.71 (0.31, 1.60)
Frequency of accompanying others to water sources			
Never		1[a]	1[a]
Sometimes		2.53(1.11, 5.80)**	2.60 (1.12, 6.01)**
All the time		5.79(2.49, 13.49)***	5.91 (2.51, 13.90)***

Table S2: *Cont*

Individual-level determinants	Models		
	**Null model AOR (95%Cl)**	**Model I AOR (95%Cl)**	**Model II AOR (95%Cl)**
Wealth Index			
Richest		1[a]	1[a]
Poorest		1.08 (0.63, 1.84)	1.06 (0.62, 1.80)
Poorer		1.10 (0 .66, 1.85)	1.10 (0.65, 1.85)
Middle		1.55 (0 .91, 2.65)	1.54 (0.90, 2.63)
Richer		1.04 (0.62, 1.73)	1.05 (0.63, 1.76)
Distance from water sources			
More than 2 km		1[a]	1[a]
Less than 1 km		3.98 (2.57,6.16)***	3.17 (1.47, 6.83)**
1km to 2km		1.17 (0.79, 1.74)	1.04 (0.63, 1.72)

Null model: intercept only model (a model without the explanatory variables)

Model I: Model with individual-level determinants with random intercept

Model II: Model with individual-level determinants with random intercept and random-coefficient for distance of the households from water sources

1[a]: reference category; AOR: Adjusted odds ratio; CI: 95% confidence interval; ** p-value < 0.005 significant and *** p-value < 0.001 strongly significant

[a]ICC Intra-class Correlation Coefficient; [a]AICAkaike's information criterion; [a]BIC Bayesian information criterion
